# Front-line Perspectives from Aging Services Providers on the COVID-19 Pandemic and Older Community Residents

**DOI:** 10.1093/geroni/igaa057.3468

**Published:** 2020-12-16

**Authors:** Victoria Raveis, Anderson Torres, Jane Arce-Bello, Donna Atmore-Dolly, Allison Nickerson

**Affiliations:** 1 New York University, Maplewood, New Jersey, United States; 2 R.A.I.N. Total Care, Inc., Bronx, New York, United States; 3 Regional Aid for Interim Needs, Inc., Bronx, New York, United States; 4 Allen Community Non-profit Programs, Jamaica, New York, United States; 5 LiveOn NY, New York, New York, United States

## Abstract

Older urban residents’ daily lives have been broadly impacted by the COVID-19 pandemic. Rapid acceleration of COVID-19 cases in mid-March 2020, forced the shuttering of senior centers in New York City’s (NYC) five boroughs. Center programming was suspended, including congregate meals for older adults. As centers, a focal point for social interaction, closed, older community residents experienced social isolation, compounded further by social distancing. Key informant interviews with the leadership of community-based multi-service agencies, provided frontline perspectives on essential programmatic services NYC’s oldest residents needed, challenges encountered, and strategies implemented to adjust to issues emerging from the pandemic. Alternative meal delivery plans were devised by the City and aging services agencies initiated wellness outreach calls and also facilitated access to technological resources to maintain social connections. Various advocacy and policy-relevant insights emerged from these experiences. Specifically, a priority in preparedness planning should be on solutions addressing the social isolation precipitated by the pandemic that older adults experienced. Technology may help. Devoting more programming to activities enabling older adults to utilize accessible technological resources is key. Efforts implemented during the pandemic have demonstrated that with preparation and access to resources, virtual communication is a feasible option for the older population. With limited access to alternative neighborhood food sources, devising a contingency plan for adequate food delivery services to replace congregate meals also merits attention. Overall, the pandemic’s widespread impact highlights the importance of creating an infrastructure that builds upon older persons’ strengths, while addressing their vulnerabilities, enabling them to thrive.

